# Safety and efficacy of a standardized intracameral combination of mydriatics and anesthetic for cataract surgery in type-2 diabetic patients

**DOI:** 10.1186/s12886-020-01343-x

**Published:** 2020-03-03

**Authors:** Marc Labetoulle, Anders Behndig, Marie-José Tassignon, Rudy Nuijts, Rita Mencucci, José Luis Güell, Uwe Pleyer, Jacek Szaflik, Paul Rosen, Alain Bérard, Frédéric Chiambaretta, Béatrice Cochener-Lamard, on behalf of the Intracameral Mydrane (ICMA), Ethics Group, C. Aguiar, J. Alio, S. Allieu, A. Behndig, G. Beltram, S. E. Benmoussa, A. Berard, P. Bonicel, J. M. Bosc, P. Bouchut, T. Bourcier, C. Boureau-Andrieux, J.-M. Buffet, F. Chiambaretta, B. Cochener, I. Cochereau, J. Colin, J. Costa Vila, M. Daghbouche, B. Delemazure, N. Duquesne, F. Fasce, J. Fernandez, O. Findl, N. Francoz, P. Gain, P. Gohier, G. Grabner, J. L. Güell, D. Hartani, S. Jaulerry, A. Kampik, M. Labetoulle, I. Lanzl, L. Laroche, C. G. Laurell, S. Lazreg, B. Le Bot, T. Lebrun, P. Lenoble, F. L’Herron, A. Liekfeld, A. Limao, M. Lobo, F. Malacaze, C. Mazit, U. Menchini, R. Mencucci, M. Mercie, E. Mertens, M. Meziane, D. Milea, S. Mohabeddine, M. Muraine, A. Muselier, F. Normand, R. Nuijts, J.-M. Perone, C. Pey, P.-J. Pisella, U. Pleyer, S. Pourjavan, P.-Y. Robert, P. Rosen, P. Rozot, N. Salaun, G. Sallet, A. Smaili, J. Szaflik, M.-J. Tassignon, J. Torras, J. M. Trigo, J. Uzzan, M. Weber, W. Williamson

**Affiliations:** 1 Service d’Ophtalmologie, Hôpital Bicêtre, APHP, Université Paris Sud, Le Kremlin-Bicêtre, 94275 Paris, France; 2 Umeå University Hospital, Umeå, Sweden; 3 Universitair Ziekenhuis Antwerpen, Edegem, Belgium; 4 University Eye Clinic, Maastricht University Medical Centre, Maastricht, the Netherlands; 5 A.O.U. Careggi, Clinica Oculistica, Università degli Studi di Firenze, Florence, Italy; 6 Instituto Microcirugía Ocular (IMO), Barcelona, Spain; 7 Universitäts-Augenklinik, Charité Campus Virchow-Klinik, Berlin, Germany; 8 Department of Ophthalmology, Medical University of Warsaw, Warszawa, Poland; 9 Oxford Eye Hospital, Oxford, UK; 10 Hôpital privé Les Franciscaines, Nîmes, France; 11 Hôpital Gabriel Montpied, CHU de Clermont-Ferrand, Clermont-Ferrand, France; 12 CHU Morvan, Brest, France

**Keywords:** Anesthetics, Cataract surgery, Diabetes, Intracameral, Mydrane, Mydriatics

## Abstract

**Background:**

Cataract surgery in diabetics is more technically challenging due to a number of factors including poor intraoperative pupil dilation and a higher risk of vision threatening complications. This study evaluates the safety and efficacy of an intracameral combination of 2 mydriatics and 1 anesthetic (ICMA, Mydrane) for cataract surgery in patients with well-controlled type-2 diabetes.

**Methods:**

Post-hoc subgroup analysis of a phase 3 randomized study, comparing ICMA to a conventional topical regimen. Data were collected from 68 centers in Europe and Algeria. Only well-controlled type-2 diabetics, free of pre-proliferative retinopathy, were included. The results for non-diabetics are also reported. The primary efficacy variable was successful capsulorhexis without additional mydriatic treatment. Postoperative safety included adverse events, endothelial cell density and vision.

**Results:**

Among 591 randomized patients, 57 (9.6%) had controlled type 2 diabetes [24 (42.1%) in the ICMA Group and 33 (57.9%) in the Topical Group; intention-to-treat (ITT) set]. Among diabetics, capsulorhexis was successfully performed without additional mydriatics in 24 (96.0%; modified-ITT set) patients in the ICMA Group and 26 (89.7%) in the Topical Group. These proportions were similar in non-diabetics. No diabetic patient [1 (0.5%) non-diabetics] in the ICMA Group had a significant decrease in pupil size (≥3 mm) intraoperatively compared to 4 (16.0%; modified-ITT set) diabetics [16 (7.3%) non-diabetics] in the Topical group. Ocular AE among diabetics occurred in 2 (8.0%; Safety set) patients in the ICMA Group and 5 (16.7%) in the Topical Group. Endothelial cell density at 1 month postoperatively was similar between groups in diabetics (*P* = 0.627) and non-diabetics (*P* = 0.368).

**Conclusions:**

ICMA is effective and can be safely used in patients with well-controlled diabetes, with potential advantages compared to a topical regimen including reduced systemic risk, better corneal integrity and reduced risk of ocular complications.

**Trial registration:**

The trial was registered at (reference # NCT02101359) on April 2, 2014.

## Background

The global prevalence of diabetes is estimated to reach 592 million by 2035 [1]. This figure represents a 64% increase in diabetics from 2013 levels, with a rate in the adult population of approximately 10% in Europe and 9% in the USA [1–3]. The association of diabetes and cataracts is well documented, with a likelihood of developing cataract 2 to 5 times higher at a younger age, in patients with diabetes mellitus compared to non-diabetic patients [2–4]. Hence, the steady increase in the prevalence of diabetes in the general population will cause an increasing surgical burden of diabetic patients requiring cataract surgery [1]. Diabetics present a number of challenges for the cataract surgeon including miosis and poor pupillary dilation intraoperatively, epithelial and endothelial dysfunction and the potential for longer surgical times and higher risk of vision threatening complications such as posterior capsule rupture and macular oedema [4–6]. Hence, achieving a large, stable pupil in diabetics is optimal for mitigating these challenges.

Intracameral (IC) mydriatic delivery was developed to address some of the drawbacks of mydriatic eye drops and the safety and efficacy has been well documented [7–9]. Mydrane (Laboratoires Théa, Clermont-Ferrand, France) is a commercially available preservative-free ophthalmic combination of two mydriatics (tropicamide 0.02% and phenylephrine 0.31%) and one anesthetic agent (lidocaine 1%) for IC administration (ICMA), just prior to beginning cataract surgery [10]. A phase 3 clinical trial reported that ICMA is safe and effective for initiating and maintaining intraoperative mydriasis and analgesia in routine cataract surgery [10]. Additionally, pupil size after ICMA administration remained stable and was statistically larger at the end of surgery compared to a topical regimen [11]. If confirmed, these characteristics may be advantageous in the diabetic population to limit the risk of complications. Despite the association of diabetes and cataracts and challenges posed in cataract surgery, there are no studies on the efficacy and safety of ICMA in type 2 diabetics undergoing cataract surgery.

To investigate the performance of ICMA in diabetic patients, a post-hoc subgroup analysis of diabetics enrolled in the phase 3 study was performed. The current paper analyzes the efficacy and safety of ICMA in patients with well-controlled type 2 diabetes in comparison to non-diabetics.

## Methods

The phase 3 study was a multicenter, international, randomized, parallel-group comparison of the safety and efficacy of ICMA (Mydrane) to a standard regimen of topical drops for cataract surgery [10]. The current paper presents a post-hoc subgroup analysis of the phase 3 study to assess the efficacy and safety of ICMA (ICMA Group) versus standard preoperative eye drops regimen (Topical Group) in patients with stable type 2 diabetes. For general comparison, the results for non-diabetic patients are also reported. This study adhered to guidelines for Good Clinical Practice. Additionally, this study adhered to the tenets of the Declaration of Helsinki. Prior to enrolling patients, ethics committee approvals were received from each country. Before participating in the study, each patient was required to sign a written informed consent document. The local health regulations were followed in each country where patients were recruited. The trial was registered at http://clinicaltrials.gov (reference # NCT02101359).

### Patients and inclusion criteria

Inclusion criteria for the phase 3 study have been previously published [10]. Patients with poorly controlled or insulin-dependent diabetes were excluded. The most current medical records were assessed for signs of well-controlled diabetes including documented laboratory tests. Only patients with well-controlled type 2 diabetes mellitus were included in the study. Diabetic patients with signs of proliferative or pre-proliferative retinopathy (using slitlamp fundus examination), or signs of macular edema (retinal thickness using optical coherence tomography) were excluded. During the selection visit, preoperative pupil dilation of at least 7 mm had to be confirmed within 30 min of instilling a standard topical mydriatic regimen. Only one eye per patient was included in this study.

### Administration of Study Medications

All investigators involved in this study had been selected on the basis of their experience (high volume cataract surgeries for several years) and their acceptance of being videorecorded for the purpose of masked analysis of the pupil size and other endpoints. Additionally, the randomization (ICMA vs. topical eye drops) was performed just before the surgery in order to keep the groups comparable (no possibility of changing the surgeon at the last moment), which was verified (comparable numbers of patients randomized in each group for a given investigator). Mydrane (Laboratoires Théa, Clermont-Ferrand, France) is a commercially available, injectable solution of 1% lidocaine combined with 0.31% phenylephrine and 0.02% tropicamide. Patients in the ICMA Group were injected with 200 μL of ICMA in the anterior chamber just after the first corneal incision. In the Topical Group, the patients received 1 drop each of tropicamide 0.5% and phenylephrine 10%, repeated 3 times at 10-min intervals beginning 30 min before surgery. Preoperatively, both groups received the same regimen of tetracaine 1% (1–2 drops at 5 and 1 min before surgery). Duovisc (Alcon Inc., Fort Worth, TX, USA) viscoelastic was used for all surgeries.

### Measurements

As previously described [10] all surgeries were videorecorded and pupil size measurements were performed by independent, trained personnel masked to the type of treatment (ICMA or topical regimen). Pupil size measurements were performed at 5 different intervals over the duration of surgery, as previously described [10]. Data were collected on the use of additional topical or intracameral medications preoperatively or intraoperatively for mydriasis or anesthesia. Postoperative visits were scheduled at 1 day, 1 week and 1 month postoperatively.

### Efficacy variables

The ability to perform capsulorhexis without additional mydriatic treatment was the primary efficacy variable. A supplementary efficacy variable was the ability to perform capsulorhexis without using additional mydriatics and a pupil size at least 6.0 mm before capsulorhexis. Additional mydriatic treatments were pupillary expansion device and/or instillation of extra medication(s) for mydriasis (for example, extra drops of phenylephrine, cyclopentolate and/or tropicamide) that were not included in the clinical trial protocol, between the initial delivery of the intracameral or topical regimen and capsulorhexis. Other assessments included the change in pupil diameter and the number of patients with no sensation of ocular pain or pressure at the various stages of surgery. Data were collected on total surgical time and the time from capsulorhexis to the end of surgery. Each surgeon noted his/her subjective assessment of the different stages of surgery, using a published scale [10].

### Postoperative safety assessments

Evaluation of postoperative safety included, measurement of best-corrected visual acuity (BCVA), ocular symptoms, slit lamp biomicroscopy and endothelial cell counts (ECC; at designated study sites based on availability of a specular microscope), funduscopy and systemic and ocular adverse events (AE). AEs were coded using the Medical Dictionary for Regulatory Activities (MedDRA) [12] (as defined in the clinical study protocol) and their severity graded using the following scale: mild - visible to the subject, but did not need any additional treatment and did not interfere with the subject’s daily activities; moderate - troublesome, could require an additional treatment, but did not interfere with the subject’s daily activities; severe - intolerable, could require an additional treatment or a modification of this treatment, could interfere with the subject’s daily activities.

### Statistical analyses

Data from the modified intention-to-treat (mITT) set were analyzed as previously described [10]. Analysis of safety included data from on all patients who received the study medications (Safety set). Data on anesthesia evaluation were included if the patient received no additional anesthetic prior to beginning surgery (mITT-An set). In the intention-to-treat (ITT) set, patients were excluded if they had treatment that could have affected mydriasis. Mainly descriptive statistics are reported. Between-group comparisons were performed with the Cochran-Mantel-Haenzel (CMH) tests based on modified ridit scores and analysis of variance. *P* < *0.05* indicates statistical significance.

## Results

In the phase 3 study, 57 (9.6%) patients with controlled type 2 diabetes mellitus were included among the 591 randomized patients. Among the diabetics, 24 (42.1%) were randomized to receive ICMA and 33 (57.9%) to receive the standard topical regimen (ITT set). The mITT set was comprised of 54 diabetics (ICMA group: 25 patients; Topical group: 29 patients), the mITT-An set of 50 (23 and 27, respectively) and the Safety set of 55 (25 and 30, respectively). One patient randomized to the Topical Group (ITT) actually received ICMA and was included in the ICMA group for statistical analyses (mITT and Safety set). Table 1 presents the preoperative patient data for diabetics and non-diabetics (ITT set).

**Table 1:** Preoperative characteristics of diabetics and non-diabetics who received intracameral ICMA or a topical regimen for cataract surgery (ITT set)

	Diabetics		Non-Diabetics	
	ICMA Group (*N* = 24)	Topical Group (*N* = 33)	ICMA Group (*N* = 271)	Topical Group (*N* = 263)
Gender				
Male, n (%)	15 (62.5)	18 (54.5)	105 (38.7)	116 (44.1)
Female, n (%)	9 (37.5)	15 (45.5)	166 (61.3)	147 (55.9)
Age (years)				
n	24	31	251	255
Mean ± SD	70.4 ± 9.2	72.2 ± 8.0	69.1 ± 9.5	70.4 ± 9.3
Min – Max	48–85	52–84	43–87	34–88
Time since cataract diagnosis (months)				
n	24	31	252	255
Median	4.2	2.8	4.3	4.2
Min – Max	0.3–134.7	0.2–57.5	0.0–636.6	0.0–111.9
Visible iris diameter at selection (mm)				
n	23	32	263	255
Mean ± SD	11.5 ± 0.4	11. 8 ± 0.5	11. 9 ± 0.6	11.9 ± 0.5
Min – Max	10.9–12.5	10.7–12.7	10.0–14.0	10.0–13.5

*SD* standard deviation, *n* number of patients, *Min* minimum, *Max* maximum, ICMA Group = patients who received an intracameral injection of a standardized combination of 1% lidocaine, 0.02% tropicamide, and 0.31% phenylephrine immediately after the 1st corneal incision; Topical Group = patients who received a topical regimen of 1 drop each of 10% phenylephrine and 0.5% tropicamide

### Efficacy of Mydriasis

**Fig. 1:**
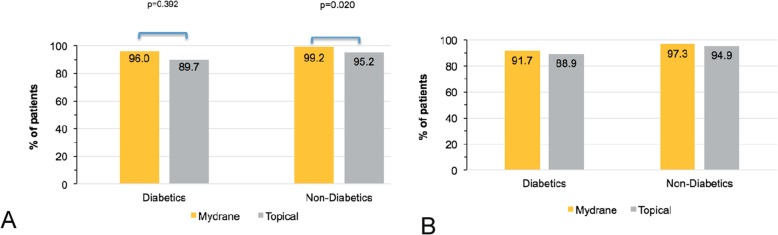
Efficacy of mydriasis in diabetic and non-diabetic patients (mITT set) who received ICMA for intracameral injection at the beginning of cataract surgery (Mydrane) or a standard topical regimen (Topical) for cataract surgery. **a** Successful capsulorhexis without any additional mydriatic treatment (Primary efficacy variable). **b** Successful capsulorhexis without any additional mydriatic treatment and a pupil size of at least 6 mm (Supplementary efficacy variable). ICMA = intracameral combination of two mydriatics and one anaesthetic; *p* < 0.05 is statistically significant

Among diabetics, capsulorhexis was performed without additional mydriatic treatments in 96.0% (24/25 patients, mITT set) in the ICMA Group and 89.7% (26/29) in the Topical Group (Fig. 1a). Data were missing in some cases due to technical difficulties with the videorecorders or poor quality video that precluded measurement of pupil size. Capsulorhexis without the use of additional mydriatics and with a pupil size ≥6.0 mm was achieved in 91.7% (22/24 patients, mITT set, data missing for 1 patient) of diabetics in the ICMA Group versus 88.9% (24/27, data missing for 2 patients) in the Topical Group (Fig. 1b). These outcomes were similar among non-diabetics.

**Fig. 2:**
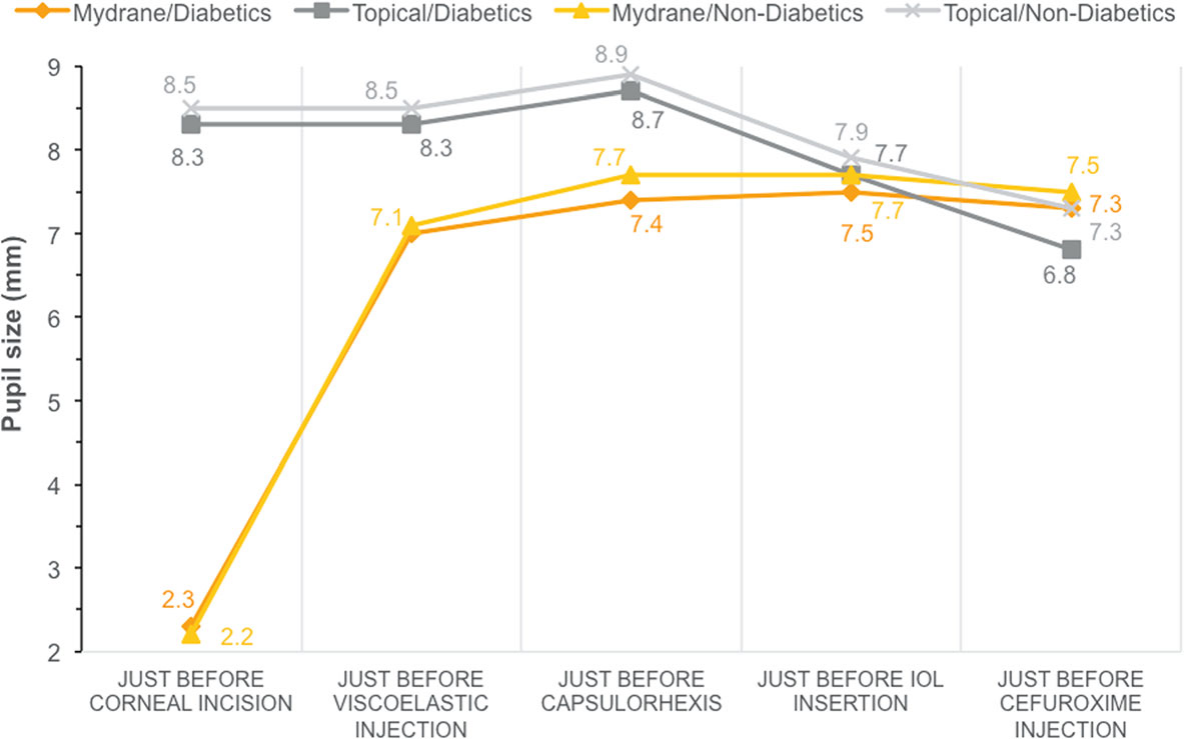
Pupil size throughout cataract surgery among diabetics and non-diabetics (mITT set) who received ICMA for intracameral injection at the beginning of cataract surgery (Mydrane) or a standard topical regimen (Topical) for cataract surgery. IOL = intraocular lens; ICMA = intracameral combination of two mydriatics and one anaesthetic

In the ICMA Group, the mean pupil size was unchanged in diabetics from just before capsulorhexis (7.4 ± 1.1 mm; range: 5.0 to 10.1 mm) to the end of surgery (7.3 ± 1.1 mm; range: 5.2 to 9.6 mm) (Fig. 2). Although higher just prior to capsulorhexis in the Topical Group (8.7 ± 0.8 mm; range: 6.7 to 10.1 mm; *P < 0.001* between groups), the pupil size decreased in this group to 6.8 ± 1.3 mm (range: 4.8 to 9.7 mm; *P = 0.109* between groups) at the end of surgery. Non-diabetics in both groups showed similar trends.

### Stability of the pupil size

**Fig. 3:**
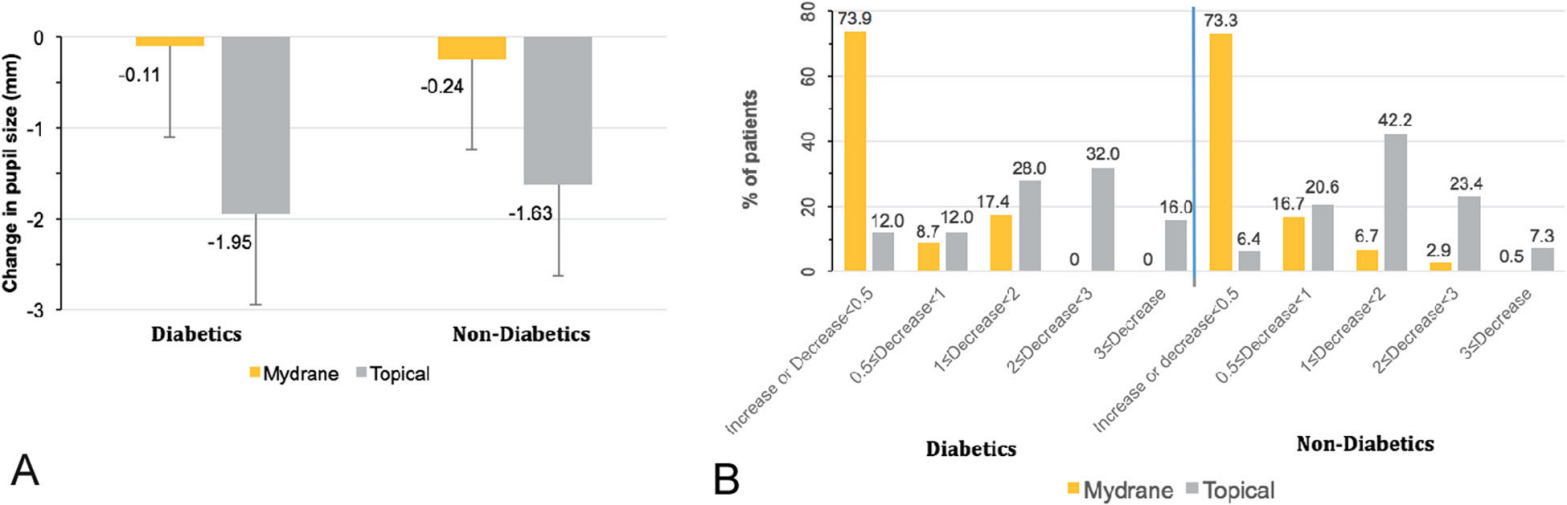
**a** Intraoperative change in pupil diameter starting just before capsulorhexis to the end of surgery among diabetic and non-diabetic patients (mITT set) who received ICMA for intracameral injection at the beginning of cataract surgery (Mydrane) or a standard topical regimen (Topical) for cataract surgery. **b** Stratification of change in pupil size from just before capsulorhexis to the end of surgery among diabetic and non-diabetic patients who received ICMA for intracameral injection at the beginning of cataract surgery or a standard topical regimen for cataract surgery. ICMA = intracameral combination of two mydriatics and one anaesthetic

The mean change in pupil diameter throughout surgery was smaller in diabetics and non-diabetics in the ICMA group (Fig. 3a). Among diabetics, the mean decrease in pupil size was − 0.11 ± 0.72 mm (median: 0.03 mm; range: − 1.7 to 1.1 mm) in the ICMA Group compared to − 1.95 ± 1.12 mm (median: − 1.94 mm; range: − 4.3 to − 0.4 mm) in the Topical Group from just before capsulorhexis to the end of surgery. The mean decrease in pupil size among non-diabetics was − 0.24 ± 0.72 mm (range: − 3.9 to 1.8 mm) for the ICMA Group and − 1.63 ± 0.96 mm (range: − 5.6 to 0.1 mm) for the Topical Group. Over the same intraoperative period, no clinically significant change in pupil diameter (change less than 1 mm) was noted in 82.6% (19/23 patients, mITT set, data missing for 2 patients) of diabetics [90.0% (189/210 patients) non-diabetics] in the ICMA Group compared to 24.0% (6/25, data missing for 4 patients) of diabetics in the Topical Group [27.0% (59/218) non-diabetics] (Fig. 3b). No diabetics [0.5% (1/210 patients) non-diabetics] in the ICMA group had a significant decrease in pupil size (≥3 mm) intraoperatively compared to 16.0% (4/25) of diabetics [7.3% (16/218) non-diabetics] in the Topical group.

### Efficacy of anesthetic

The proportions of patients with no ocular pain/pressure for all phases of surgery was similar among diabetics and non-diabetics in the ICMA and Topical Groups (*P* > *0.05*, all comparisons). Among diabetics, more patients in the ICMA Group (69.6%, 16/23 patients, mITT-An set) than the Topical Group (51.9%, 14/27 patients, mITT-An set; *P* = *0.321*) reported no sensation of pain just before IOL insertion (ie, during the active phase of the surgery with the most surgical maneuvers). Non-diabetics had similar outcomes [78.8% (160/203 patients) vs 69.9% (146/209), respectively; *P* = *0.067*].

### Duration of surgery

**Fig. 4:**
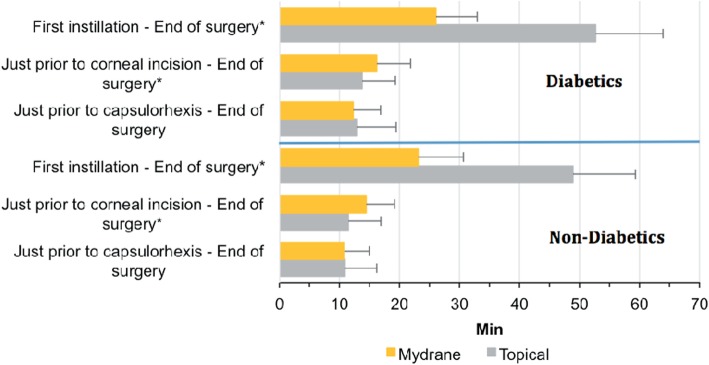
Duration of the different phases of surgery for diabetic and non-diabetic patients (mITT set) who received ICMA for intracameral injection at the beginning of cataract surgery (Mydrane) or a standard topical regimen (Topical) for cataract surgery. These phases included a mandated 1 min 30 s waiting time for the ICMA Group in the study protocol. ICMA = intracameral combination of two mydriatics and one anaesthetic

The mean duration of the active phase of surgery (just prior to capsulorhexis to the end of surgery) was similar for diabetics [ICMA Group: 12.2 (range: 6.7 to 22.0) min; Topical Group: 12.8 (range: 6.8 to 27.8) min; *P* = *0.669* between groups] and non-diabetics [10.8 (range: 3.6 to 35.8) min vs 10.9 (range: 4.2 to 66.8) min, respectively; *P* = *0.942*] (Fig. 4).

### Surgeon grading

Surgeons graded the different phases of surgery as uncomplicated for most diabetic patients in both Groups [between 88.9% (48/54 patients, mITT set) and 92.6% (50/54)] (*P* > *0.05*, all between-group comparisons). For non-diabetics, a statistically significantly lower number of slightly complicated or complicated cases during IOL implantation were reported in the ICMA Group compared to the Topical Group (2.9%, 7/243 patients vs 7.1%, 18/252 patients, respectively; *P* = *0.030*). There was no statistical between-group difference in surgeon grading among non-diabetic patients for the other phases of surgery (*P* > *0.05*, all comparisons).

### Safety

The incidence of any ocular AE among diabetics was 8.0% (2/25 patients, Safety set) in the ICMA Group and 16.7% (5/30 patients) in the Topical Group (Table 2).

**Table 2:** Adverse events in diabetics who received intracameral ICMA or a topical regimen for cataract surgery (Safety set)

ICMA Group	Topical Group
*Complications (number of patients)*	*Complications (number of patients)*
Mild conjunctival hyperaemia (1)	Mild conjunctivitis (1)
Mild punctate keratitis (1)	Moderate punctate keratitis (2)
	Moderate dry eye (1)
	Moderate eyelid ptosis (1)
	Moderate posterior capsule rupture (1)^a^
Severe transient ischaemic attack (1)	Mild type 2 diabetes mellitus (1)

ICMA Group = patients who received an intracameral injection of a standardized combination of 1% lidocaine, 0.02% tropicamide, and 0.31% phenylephrine immediately after the 1st corneal incision

Topical Group = patients who received a topical regimen of 1 drop each of 10% phenylephrine and 0.5% tropicamide. ^a^Moderate posterior capsule rupture was a limited rupture that did not require anterior vitrectomy

Among diabetics, there were no ocular AE related to study treatment in both groups. The incidence of systemic AE was 4.0% (1/25 patients) in the ICMA Group and 3.3% (1/30 patients) in the Topical Group. One serious systemic event (transient ischemic attack) was reported in the ICMA Group. None of the systemic AEs were related to the study medication.

There was no statistical between-group difference in the endothelial cell density at 1 month postoperatively among diabetics (*P* = 0.627) and non-diabetics (*P* = 0.368) (Table 3).

**Table 3:** Change in endothelial cell density from preoperatively to 1 month postoperatively (Safety set)

	Diabetics		Non-Diabetics	
	ICMA Group (*N* = 16)	Topical Group (*N* = 12)	ICMA Group (*N* = 150)	Topical Group (*N* = 156)
Preoperative Endothelial Cell Density (cells/mm^2^)				
Mean ± SD	2262.1 ± 396.3	2345.9 ± 283.5	2400.1 + 420.6	2401.9 ± 415.3
Min – Max	1330–2813	1970–2943	1498–3692	974–3662
Change in Endothelial Cell Density at 1 month post-surgery (cells/mm^2^)				
Mean ± SD	− 163.4 ± 347.5	− 121.3 ± 412.0	− 224.8 ± 417.3	− 185.2 ± 388.6
(%)	(−7.2)	(− 5.2)	(− 9.4)	(− 7.7)
Min – Max	− 955 – 526	− 749 – 708	− 1843 – 976	− 2076 – 600

A decrease in endothelial cell counts is indicated by negative numbers; ICMA Group = patients who received an intracameral injection of a standardized combination of 1% lidocaine, 0.02% tropicamide, and 0.31% phenylephrine immediately after the 1st corneal incision. Topical Group = patients who received a topical regimen of 1 drop each of 10% phenylephrine and 0.5% tropicamide

Seven diabetics who received 2 (or more) injections of ICMA had a higher change in endothelial cell density of − 375.4 ± 349.5 (range: − 955 to 31) cells/mm^2^ (− 15.8%) than patients who were injected only once. For the 39 non-diabetic patients who received 2 (or more) injections of ICMA, the change in endothelial cell density was − 318.4 ± 479.1 (range: − 1843 to 324) cells/mm^2^ (− 10.7%).

At 1 week postoperatively, none of the diabetics reported ocular pain. Among non-diabetics, ocular pain was reported at 1 week postoperatively by statistically significantly fewer patients in the ICMA Group (0.8%, 2/246 patients, Safety set) compared to the Topical Group (4.4%, 11/249 patients; *P* = *0.004*). Irritation/burning/stinging of the eye at 1 month postoperatively was reported by 8.0% (2/25) of patients in the ICMA Group and 17.2% (5/29) in the Topical Group among diabetics (*P* = *0.296*), and by statistically significantly fewer patients in the ICMA Group (4.6%, 11/241 patients) compared to the Topical Group (11.7%, 29/248 patients) among non-diabetics (*P* = *0.008*).

Postoperatively, there were no clinically significant concerns in diabetics from the ICMA and Topical groups in terms of ocular inflammation (based on assessing anterior chamber cells/flare), corneal pachymetry, retinal thickness, retinal examination and intraocular pressure. All diabetics but one benefited from cataract surgery, with visual acuity increasing by at least 3 lines in 83.3% (20/24 patients, Safety set, data missing for 1 patient) of diabetics in the ICMA Group and 90.0% (27/30 patients) in the Topical Group at last visit.

## Discussion

Cataract surgery in diabetics can be challenging due to the poor pupillary response to mydriatics [13–16]. Currently, in Western countries, 1 in 5 patients undergoing cataract surgery is diabetic [17]. The outcomes of this study indicate that ICMA was equivalent to a standard topical regimen for cataract surgery in diabetics and the efficacy of ICMA was equivalent to the use of ICMA (and topical regimen) in non-diabetic patients. ICMA injection just after the first corneal incision resulted in rapid and stable mydriasis without requiring additional mydriatics or pupil expanders in the large majority of diabetics, similar to the standard topical regimen and to non-diabetics.

A 6-mm pupil is commonly considered adequate for performing capsulorhexis [6, 11, 18–20]. In the phase 3 study, surgeons felt the pupil size after ICMA was adequate for safely performing cataract surgery [10]. Given the similar outcomes in the current study, ICMA should also be adequate for cataract surgery in patients with well-controlled diabetes.

Stable mydriasis is fundamental for cataract surgery [18]. We found that the change in intraoperative pupil size in diabetics in the ICMA Group was smaller than in the Topical Group of diabetics. Additionally, the large majority of diabetics (73.9%) in the ICMA Group experienced a decrease less than 0.5 mm in pupil size from capsulorhexis to the end of surgery. These outcomes for diabetics are comparable to previous comparisons of ICMA and topical regimen in non-diabetics [10, 11].

Intraoperative miosis with topical therapy is well documented [6, 10, 20]. This factor becomes especially important in diabetics who may have an abnormal iris response to mydriatics due to autonomic dysfunction [6], a larger [21] or more tenacious lens [16] and anterior chamber shallowing [22], which may predispose to a more technically demanding surgery. Intracameral delivery of ICMA may mitigate the risk of complications such as posterior capsular rupture in diabetics due to stable mydriasis and a pupil size that remains 7 mm or larger to the end of surgery as reported in both diabetics and non-diabetics in the ICMA Group in the current analysis. An additional advantage of using ICMA for diabetics is the reduced quantity of injected mydriatics, limiting their systemic absorption in a population with higher cardiovascular risks [10, 14].

Pain during the surgical maneuvers of cataract surgery may lead to an uncooperative patient. The presence of lidocaine in ICMA may mitigate intraocular pain during cataract surgery [23, 24]. Additionally, the use of ICMA may decrease the number of topical drops used compared to topical regimens, thus mitigating ocular surface toxicity. This is of particular interest as diabetic keratopathy is one of the numerous ocular complications associated with diabetes. The compromised integrity of the corneal epithelium and ocular surface may make diabetics more prone to corneal damage [6, 13]. Topical mydriatics and anesthetics can disrupt the corneal epithelium making the diabetic cornea more susceptible to punctate keratitis and infections [6, 13]. Impaired corneal wound healing among diabetics would further increase the risk of trophic complications [6, 13]. By reducing the mydriatic and anesthetic load on the cornea and delivering these agents to the intended site of action, ICMA may reduce the risk of postoperative corneal complications.

Longer procedures may also lead to an uncooperative patient during surgery. Based on previous experience [25], ICMA may decrease the duration of the entire procedure (preoperative care, perioperative and discharge) in actual clinical practice because it dispenses with the requirements of repeated instillation of medications well before the beginning of the procedure.

Surgeons graded surgery as mostly uncomplicated in both diabetic groups. In the current study, there were no safety concerns regarding the use of ICMA in diabetics. For example, there were no ocular or systemic events related to the study medications. The ocular AEs that occurred in the two diabetics in the ICMA Group were mild and resolved with observation without further sequelae. One serious case of transient ischemic attack occurred in one diabetic patient in the ICMA Group that was deemed by the investigator as unrelated to the study medication (occurred 6 days postoperatively and was more likely due to a history of vascular disorders, including high blood pressure and a stroke 4 years previously). Endothelial cell loss was similar in diabetics and non-diabetics. This loss was well within the range (9 to 11%) reported for non-diabetics undergoing cataract surgery with the standard topical regimen for anesthesia and mydrasis [26, 27]. However, the small sample of patients that underwent endothelial microscopy warrants judicious interpretation of this outcome. Endothelial cell loss was greater in patients who received 2 or more injections of ICMA in both diabetics and non-diabetics. During the phase 3 study, surgeons were allowed to deliver more than one injection of ICMA if they deemed the pupil size was inadequate. A lack of experience with ICMA and the early learning curve for ICMA was the most common reason for the delivery of the additional doses. Masked analysis of the surgical videos indicated that the extra injections offered no clinically significant value. Subsequent approval and the product monograph states that only one injection of ICMA is recommended as greater dilation is not achieved with additional doses [11]. At last visit postoperatively, there were no clinically significant concerns related to ICMA in diabetics.

This study has some limitations. The small sample size, the post-hoc analysis and the absence of stratification from the inception of the study for diabetics resulted in insufficient power to determine subtle differences between diabetics in the ICMA and Topical Groups. However, the randomized nature of the overall study mitigated bias in the outcomes. Additionally, we found no clinically significant differences in using ICMA in diabetic compared to non-diabetic patients. Surgeon experience was likely not a factor in the outcomes as the same surgeons performed surgery in diabetics and non-diabetic patients, hence surgeon seniority, experience and skill should affect both groups equally.

## Conclusions

Analysis of the diabetic subpopulation in this phase 3 study indicates that intracameral administration of ICMA just after the first corneal incision is an effective and safe alternative to the routine topical regimen for initiating and maintaining intraoperative mydriasis during cataract surgery in diabetic patients. Additionally, the anterior chamber structures in a diabetic eye (without significant diabetic retinopathy) seem to respond similarly to the normal non-diabetic anterior chamber structures after injection of ICMA. Administration of ICMA should only be performed in patients (including diabetics) who have previously demonstrated satisfactory pupil dilation with topical mydriatics. It can be safely used in patients with controlled diabetes, with potential advantages compared to the conventional eye drop regimen regarding reduced systemic risk, better corneal integrity and reduced risk of ocular complications.

## Data Availability

Data available on request Marc Labetoulle MD, PhD. Service d’Ophtalmologie, Hôpital Bicêtre, APHP, Université Paris Sud. 94275 Le Kremlin-Bicêtre, France. E-mail: marc.labetoulle@bct.aphp.fr; Phone: 33 1 45 21 36 90.

## Abbreviations

AE: Adverse event
BCVA: Best-corrected visual acuity
CMH: Cochran-Mantel-Haenzel
ECC: Endothelial cell count
IC: Intracameral
ICMA: Intracameral combination of 2 mydriatics and 1 anesthetic
ITT: Intention-to-treat
mITT: Modified intention-to-treat
mITT-An: Modified intention-to-treat anesthesia set
